# Book Review

**DOI:** 10.1054/bjoc.2000.1685

**Published:** 2001-04


*British Journal of Cancer* (2001) **84**(7), 1008–1009
© 2001 Cancer Research Campaign
doi: 10.1054/ bjoc.2000.1685, available online at http://www.idealibrary.com on IDEAL®
http://www.bjcancer.com

### Book review

### Cancer: The Evolutionary Legacy

Mel Greaves, Oxford University Press, 2000
Price: £19.99, $27.50

The most common question asked to cancer physicians must be: Why me? In this very well written book, Mel Greaves argues that most adult cancers are a result of a discordance between our inherent biology and our behaviour. In a truly entertaining and fascinating read, he uses Darwinian reasoning to address all the commonly asked questions: Why does a healthy person get cancer? Why do different cancer types predominate in different ethnic and social groups? Why does treatment sometimes work so well? And why does the incidence of cancer appear so high during the last century?

Apart from some acknowledged, highly speculative theory, most of his views are less a radical revision than a reminder. Nevertheless, Greaves has the ability to express this reminder in a very eloquent language, with a vast knowledge including fascinating anecdotes and a broad understanding of current trends in cancer research and therapies.

The book is divided into four sections (and 25 chapters). After each section is a 'Notes' chapter, which is packed with facts and references for those interested in expanded detail of concepts discussed.

Part One provides a background to what cancer is, historical case descriptions (including probable cases of Gorlin's syndrome in Egyptian mummies!), and introduces 'the social ratchet'. His social ratchet is our ability to survive long after our natural reproductive period and our ability (and will) to interfere with our biology and therefore disturb the equilibrium established by our million-year-old evolutionary legacy.

Part Two explains basic properties of cancer cells, including clonality, mutations of oncogenes and tumour suppressor genes, telomerase activity and progressive steps of carcinogenesis. These chapters are written in a style to be mostly understood by non-scientists.

Genetic mutations have driven the evolution of all living organisms and accelerated mutations and selection of the fittest also drive the development of a cancer clone. Greaves refers to Manfred Eigen's *Steps towards life: a perspective on evolution*, to explain the concept that mutants with superiority over their precursor 'wild-type' forms, provide a selective advantage and an expanded basis for further selective forces.

A theme emerges, and is discussed in the context of observations in 1926 and 1893 by Morley Roberts (*Malignancy and Evolution*) and Herbert Snow respectively, that cancer clones derive from stem cells that behave like evolutionary remnants: i.e. retain the memory of the selfish behaviour of our unicellular ancestors. Greaves cleverly coins the contemporary phrase 'back to the future' to refer to this progression from a tissue restraint cell with finite replicative potential to an immortal cell with the capacity for territorial expansion and in which proliferation is uncoupled from differentiation and its functional role.

Part Three mainly deals with specific types of cancer and their origins. These eleven chapters are very easy to read and difficult to put down when started. Chapter 14 (And then you set fire to it?) deals with smoking and cancer. It covers the intriguing history of smoking, explaining where terms like *Nicotiana tabacum* originated from, and includes a viscious, yet justifiable attack on those promoting and profiting from the industry (including the current British Royal Family).

Chapters 15 and 16 deal with 'Womens' and Mens' Troubles' respectively. Breast cancer risks and causes are discussed in the context of a comparison between 'modern women' and 'primitive Eve'. Primitive Eve's menarche was later, but age of first pregnancy much earlier. The parity and length of breast-feeding was also much higher and longer in primitive Eve, resulting in a marked reduction in number of ovulatory cycles compared to modern women. This forms the basis for his arguments on the causes of breast and ovarian cancer.

Humans have the largest prostate of all primates. Greaves suggests that Darwinian selection would have favoured males whose prostate lubrication (for sperm flow and fertilization) was ready at all times (explaining the size of our prostates). I would have thought that this reasoning and selective pressure would have favoured large prostate development in some of the other mammals too? He further hypothesizes that testosterone levels, unlike oestrogen of females, do not decline rapidly after the 'normal' reproductive age, and that sexual activity also provides regular boosts to pro-prostate proliferating proteins like PSA and testosterone. Greaves therefore proposes that our uncoupling of sex from reproduction (and perhaps unbalanced diets) are the main culprits triggering prostate cancer. Although this is clearly an over-simplified vision of the pathogenesis of prostate cancer, it does make you think, and in many parts of the book, Greaves achieves just that: his reasoning and speculations are wonderful points for debate and argument. I am sure Greaves intended this when overstating (and occasionally expressing tongue-in-cheek) his own theories like that of the origin of prostate cancer.

The pygmy chimpanzee or bonobo (Democratic Republic of Zaire) also mates regularly, (and in the missionary position) for pleasure, and not just during female ovulation (*The Evolution of Human Sexuality*, by Malcolm Potts and Roger Short, Cambridge University Press, 1999). I am sure our bipedal primate forefathers enjoyed sex as much as we do, but perhaps they, like the bonobo, also would have (or did) develop prostate cancer if they lived long enough.

My own view is that prostate cancer is the result of organ-specific differences in genome maintenance mechanisms, including increased mutational rates with age in prostatic cells and an age-associated decrease in the repair of DNA damage. Testosterone surges may contribute to the proliferative potential of mutated cells. This alone would of course not explain the clear ethnic variation in incidence, but diet and infection (possible yet-to-be-discovered microorganism) could be further contributory factors.

In chapter 19 (Travelling Light) Greaves tackles the causes of skin cancer, and why evolution would have favoured us to end-up with very light or very dark skins? Why have modern humans (most of us anyway) lost the majority of our body hair? Was it to avoid self-ignition around fires, or, as Desmond Morris suggested in *Naked Ape* to facilitate heat-loss in a hunter in pursuit of his prey? Presumably, the hunters then sexually preferred less-hirsute women resulting in a founder effect. This chapter is again a fascinating read, which conveys many known and much less known facts including descriptions of likely cases of metastatic melanomas in 2400-year-old Inca skulls.

Chapter 20 (The Great Glut) deals with intestinal tumours, and as expected the evils of our modern diets are entertainingly discussed. He also reminds us of probable carcinogens in barbecued red meat. I always had a problem with this hypothesis, as some of our cave-living hominid ancestors, who just acquired the ability to light fire, surely indulged in roasted meat? However, we should listen to his advice and at least have some celery when next invited to a barbeque.

Part Four covers current and future therapies and preventative measures. As most epithelial cancers result at least partly from our social ratchet of pleasurable behaviour – sex, smoke and sun, food and drink – it is difficult to see if (and indeed why) we could/should radically alter this conflict between biological evolution and behaviour. Greaves goes on to discuss other future options in the prevention and treatment of cancer.

I would highly recommend this book to any scholar of cancer research or cancer medicine. It is written in a style and lucidity that also makes it an accessible read to non-scientists, patients and their families. Mel Greaves provides anyone interested in cancer with a real treat of facts, speculations and rare anecdotes, to explore the origin of cancer.

*Chris Boshoff*
*Wolfson Institute for Biomedical Research*
*Cruciform Building*
*University College London*
*WC1E 6BT*
*c.boshoff@ucl.ac.uk*

*© 2001 Cancer Research Campaign*

